# Mechanism for High-Precision Control of Movement at Maximum Output in the Vertical Jump Task

**DOI:** 10.3390/e26040300

**Published:** 2024-03-28

**Authors:** Hiroki Murakami, Norimasa Yamada

**Affiliations:** Graduate School of Health and Sport Sciences, Chukyo University, 101 Tokodachi, Kaizu-cho, Toyota 470-0393, Aichi, Japan; nyamada@sass.chukyo-u.ac.jp

**Keywords:** speed and accuracy, trajectory, maximum effort, whole-body movement

## Abstract

Human movements are governed by a tradeoff between speed and accuracy. Previous studies that have investigated the tradeoff relationship in sports movements involving whole-body movements have been limited to examining the relationship from the perspective of competition-specific movements, and the findings on whether the relationship is valid have not been unified. Therefore, this study incorporated a vertical jump task with the introduction of a condition in which landing position control was added to evaluate the essence of a sports movement that requires both speed and accuracy. Accuracy was examined using a method that quantifies the coordinates of the landing and takeoff positions using entropy. The mechanism of that tradeoff was then examined by confirming the phenomenon and analyzing the 3D vector trajectories. An increase in accuracy and a decrease in speed were observed when the landing position was the control target, even in the vertical jumping task normally performed at maximum effort, and the 3D velocity vector was characterized by the following: a reduced scalar and a more vertical direction. While the entropy from the takeoff to the landing position seemed to decrease when the accuracy of the landing position improved, the following noteworthy results were obtained given the characteristics of the vertical jump. Unlike traditional feedback control in the entropy reduction in hand movements, the trajectory is predetermined in a feedforward-like manner by controlling the initial velocity vector at takeoff, which allows the landing point to be adjusted.

## 1. Introduction

Human movements are governed by a speed and accuracy tradeoff [1]. Based on Woodworth’s experimental [2] results on hand movements, Fitts [3] developed a strong relationship between speed and accuracy in experiments on repetitive hand movements using a stylus. This relationship was systematized from the experimental results in which movement time (MT) increased linearly with increasing task difficulty (ID). In addition, because an increase in ID indirectly implies a demand for higher accuracy, the relationship (speed and accuracy tradeoff) can be read from the experimental results as MT increases (average speed decreases) as the demand for accuracy increases. Furthermore, Fitts formalized the relationship between speed and accuracy by linking it to Shannon’s [4] information theory ideas. Fitts’ experimental results, also called “Fitts’ law”, are recognized as an important law that describes the characteristics of human physical movement [5]. In addition, pointing devices have become widely used with the development of computers, and it is necessary to evaluate and predict their performance prior to the selection and design of input devices and interfaces, to which Fitts’ law has contributed. This is an application of Fitts’ law in the field of human–computer interaction (HCI), and is a very important theoretical law for people who use various devices, such as PCs and tablets, as a matter of course. Furthermore, although the movements that lead to this law are simple, repetitive hand movements, various movements have been extended to be applicable to this law. For example, movements using a joystick or a mouse are applicable [6,7]. Research is also being conducted to determine whether this law is valid for sports movements that require whole-body movement. The following paragraphs introduce those reports, but an overview of the previous studies shows that they have not reported unified findings on whether the tradeoff relationship between speed and accuracy, which Fitts experimentally demonstrated, is established in any given case. In addition, these studies are limited to examining the relationship from the perspective of competition-specific movements; therefore, they have not been able to approach the fundamental mechanism under which the relationship is established.

The following studies have reported that this relationship is established. Etnyre [8] compared the accuracy of advanced, intermediate, and beginner dart throwers hitting the dartboard under two different conditions: throwing normally or throwing as hard as possible. The results demonstrated that accuracy was lower in the as hard as you can condition than in the normal condition for all groups. Furthermore, Tillaar and Ulvik [9] examined ball speed and accuracy from the center of a target placed at the center of a soccer goal with the dominant and non-dominant feet using soccer kicking motions. They found that ball speed decreased with the dominant foot in the accuracy instruction, whereas accuracy increased significantly. Recently, Liang et al. [10] showed that a tradeoff between speed and accuracy was observed for the throwing motion of cricketers under time constraints.

In contrast, the following studies have reported that this law does not hold. For example, studies on golf putting and soccer kicking movements have demonstrated that the success rate does not increase even if the movement time is increased (even if the average speed of the movement is decreased) [11,12]. Akbas et al. [13] examined whether external conditions of target width and target distance in fencing lunge movements (conditions related to changes in speed and accuracy) affected postural control mechanisms but found no effect.

Since previous studies have not reported unified findings on the above relationship for whole-body exercise and are limited to examining it from a competition-specific movement perspective, the following questions cannot be answered: When does the relationship occur (e.g., is it established at the beginning of the movement, near the end, or throughout the movement)? What is that underlying mechanism? This may be because the movements of many competitive sports are complex multi-joint movements, making it difficult to identify the factors involved. In a review article on handball throwing motions by Vila and Ferragut [14], the results of several previous studies indicated that this relationship may not hold true for complex movements. Schmidt [5] described one of the two exceptions to this relationship as “cases in which extremely rapid and forceful actions are involved”, but there are often situations in real sports movements where high accuracy control is required while exerting maximum speed. Although a previous study [15] dealt with handball overarm throw motion and showed how three different types of instruction (emphasis on speed, accuracy, and both) affected the kinematic variables of the throw motion through a correlation analysis of several variables, the study found that the kinematic parameters changed with different teaching methods. However, as pointed out above, the study deals with competition-specific movements and fails to approach the mechanism of the relationship between essential speed and accuracy.

To address the essential issue of speed and accuracy in whole-body movements, Yamada et al. [16] devised an experiment in which a landing position control task, which is not normally required, was added to the vertical jump movement. This was carried out in the laboratory to reproduce a situation in which a sports movement requiring both speed and accuracy is performed and to approach the mechanism of actual sports movements in which maximum effort is exerted without compromising the accuracy of the movement. In this experiment, two conditions were used: a vertical jump with maximum effort and a vertical jump with maximum effort and instructions to land at the jumped position. When the landing position was controlled, the jumping height was reduced (the vertical velocity at the takeoff point was reduced) and the accuracy of the landing position was improved, although the vertical jump was performed with the same maximum effort in both conditions. This result indicates that a tradeoff between speed and accuracy may have occurred during whole-body movements. Furthermore, they explained the necessity of examining the magnitude, direction, and duration of action of the 3D force vector to investigate the mechanism of this phenomenon and proposed an analysis method focusing on the trajectory of the 3D vector. However, that study was only a qualitative analysis using typical examples of each condition, and further studies are needed to quantitatively clarify the mechanism of the phenomenon. Recent studies [17,18] have quantified 3D trajectories by calculating the probability of coordinates at any point in time (e.g., at 50% of the trajectory or at the end point) of multiple trajectories during iterative tapping tasks, similar to Fitts, and by calculating entropy.

The analysis of these studies followed the theory that probability is the basis for information entropy [4]. Previous studies adapting entropy to human movements related to speed and accuracy were conducted using the probability of data distribution at several points in the trajectory of a simple hand-aiming motion as an extension of Fitts’ study [19,20,21]. Entropy analysis based on the coordinates of multiple time points of motion trajectories considered in our recent studies [17,18] has two advantages: (1) the ability to quantify the variation in trajectories at similar arbitrary points in time (e.g., at the end of the trajectory or at the 50% point) and to compare their values across conditions; and (2) the ability to compare the greater or lesser value of multiple quantified trajectories at any given point in time, allowing one to estimate at what part of the movement the information processing took place. In this case, a higher entropy value at a point in time indicates more variation at that point, which is useful for comparing the accuracy between conditions. In addition, a decrease in entropy (magnitude of variation) when comparing values between points in the direction that time moves forward (e.g., from point A to point B) can be considered a decrease in irregularity. Since uncertainty reduction requires information [4], more information must be processed to better reduce uncertainty (increased occurrence of information processing). In other words, the point at which the entropy of a trajectory decreases through multiple trajectory analysis indicates a transition to a more order-forming motion, and this change indicates that specific information processing has occurred. This analytical approach allows kinematics studies to compare multiple movement trajectories using entropy to gain a more detailed understanding of when and how information processing is necessary to improve accuracy and efficiency. In addition, entropy analysis has been used in studies on whole-body movements, such as sports movements, to evaluate postural stability and how athletes move around the field during games [22,23,24]. However, this method of quantifying specific intervals of hand-movement trajectories using entropy analysis has not yet been used in studies targeting whole-body movements. Therefore, based on the merits of entropy analysis of trajectories for hand movements, this study attempts to incorporate this entropy analysis as a method for quantifying the accuracy of vertical jumps.

Therefore, to approach the essence of sports movements that require both speed and accuracy, this study implements a vertical jump task that introduces a condition of adding landing position control using a method similar to that of Yamada et al. [16]. The mechanism of the speed and accuracy tradeoff was then examined by checking whether the phenomenon of that tradeoff occurs and by analyzing the trajectory.

## 2. Materials and Methods

### 2.1. Participants

The participants were 10 male students (21.2 ± 0.5 age; height: 173.8 ± 8.0 cm; weight: 64.2 ± 3.3 kg) aged 20 years or older belonging to an athletic club. All study procedures were conducted in accordance with the Declaration of Helsinki and the ethics code of Chukyo University, and were approved by the ethics committee of Chukyo University (approval number: 2021-42). The participants provided written informed consent prior to participation.

### 2.2. Apparatus

Signals from a single force plate (Bertec Corporation, Columbus, OH, USA) digitized at 1000 Hz (force data in 3 axial directions (x, y, z) and with the center of pressure (cop) in 2 axial directions (x, z)) were imported onto a PC.

### 2.3. Experimental Design

**Figure 1:**
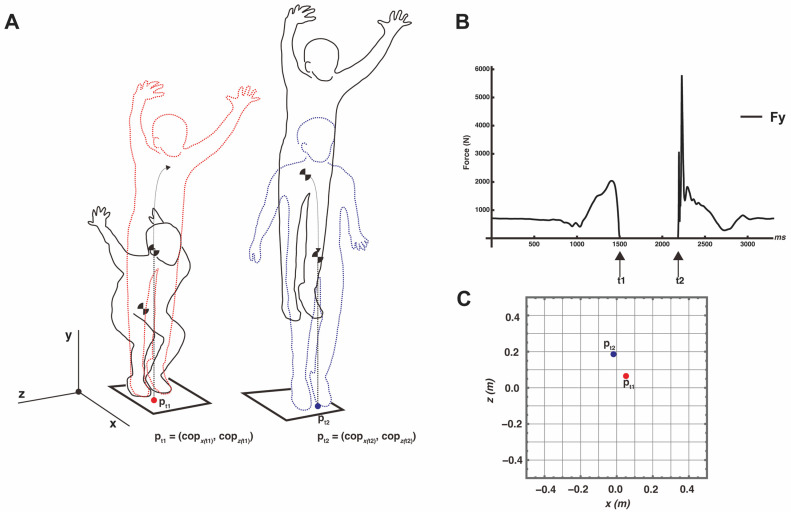
Conceptual diagram of experimental set up and primary data obtained from force plate. (**A**) A typical example of sinking (solid black line) to takeoff (red wavy line) (left) and staying in the air (solid black line) to landing (blue wavy line) (right). The coordinates of the point of takeoff (p_t1_) and landing (p_t2_) are computed from the cop data. (**B**) A typical example of force data (F_y_) obtained from a force plate. While not in contact with the force plate (i.e., in air), the value is zero. (**C**) Typical x-z plane coordinates of the takeoff and landing points. The origin is the initial value (the value at the point of rest, p_0_).

After a thorough warm-up, the participants were instructed to perform a vertical jump on a force plate under the following conditions: (1) perform the vertical jump with maximum effort (normal condition) and (2) perform the vertical jump with maximum effort and control the landing to land in the takeoff position (adjusted condition). They were not instructed to perform arm-swinging or upper-body recoil movements. They were allowed to self-report and take sufficient rest between trials. A conceptual diagram of the experiment is shown in Figure 1.

### 2.4. Experimental Procedure

Participants entered the laboratory and were briefed on the tasks and risks involved in the experiment. The experimenter gave the participants 10 min to warm up. They were free to stretch their feet. The experimental movements were announced before the warm-up; however, the experimental conditions were not explained. They performed 10 trials each in normal and adjusted conditions, in that order. The experimenter instructed them to complete a vertical jump on the force plate from a stationary position on the force plate after receiving a signal. They were not given feedback on their trials until all of the trials were completed.

### 2.5. Data Analysis

All numerical calculations, including the analyses, were performed using Mathematica 12.3.1.0 (Wolfram Research, Champaign, IL, USA).

#### 2.5.1. Calculation of the Acceleration, Velocity, and Position Vectors of the Center of Gravity

All of the data collected from the force plate (FP4060-07-TM-2000, Bertec, USA, Natural Frequency: Fx: 300, Fy: 300, and Fz: 450) were raw, and no smoothing was applied. Prior to calculating each performance variable, the following variables were calculated from force vector $F_{t}$ obtained from the force plate. $F_{t}$ is the sum of the temporal variation in the body’s movement $mv_{t}$, gravitational acceleration vector *g*, and weight multiplied by the mass *m* of the experimental participants, and is expressed according to Equation (1) as follows:

(1) $$F_{t}=m\frac{dv_{t}}{dt}+mg,g=\left(0,\;9.81,\;0\right).\;$$

In Equation (2), the acceleration vector $a_{t}$ of the center of gravity is calculated as follows:

(2) $$a_{t}=\frac{dv_{t}}{dt}=\frac{F_{t}}{m}-g.\;$$

Therefore, if the time when the movement starts is 0 and the time when the foot leaves the force plate is $t_{1}$ (takeoff time), the experiment starts from a stationary state (initial value of the velocity vector is 0), and the initial value of the center of gravity position at rest coincides with the center of pressure (cop), which is obtained from the force plate. The following equation is used to calculate the movement speed. The velocity ($v_{t}$) and position ($p_{t}$) vectors from the start of the movement to the takeoff time can be calculated according to the following equations:

(3) $$v_{t}=\int_{0}^{t_{1}}a_{t}dt+v_{0},\;v_{0}=\left(0,\;0,\;0\right).\;$$

(4) $$p_{t}=\int_{0}^{t_{1}}v_{t}dt+p_{0},\;p_{0}=\left({cop}_{x\left(0\right)},\;0,\;{cop}_{z\left(0\right)}\right).\;$$

**Figure 2:**
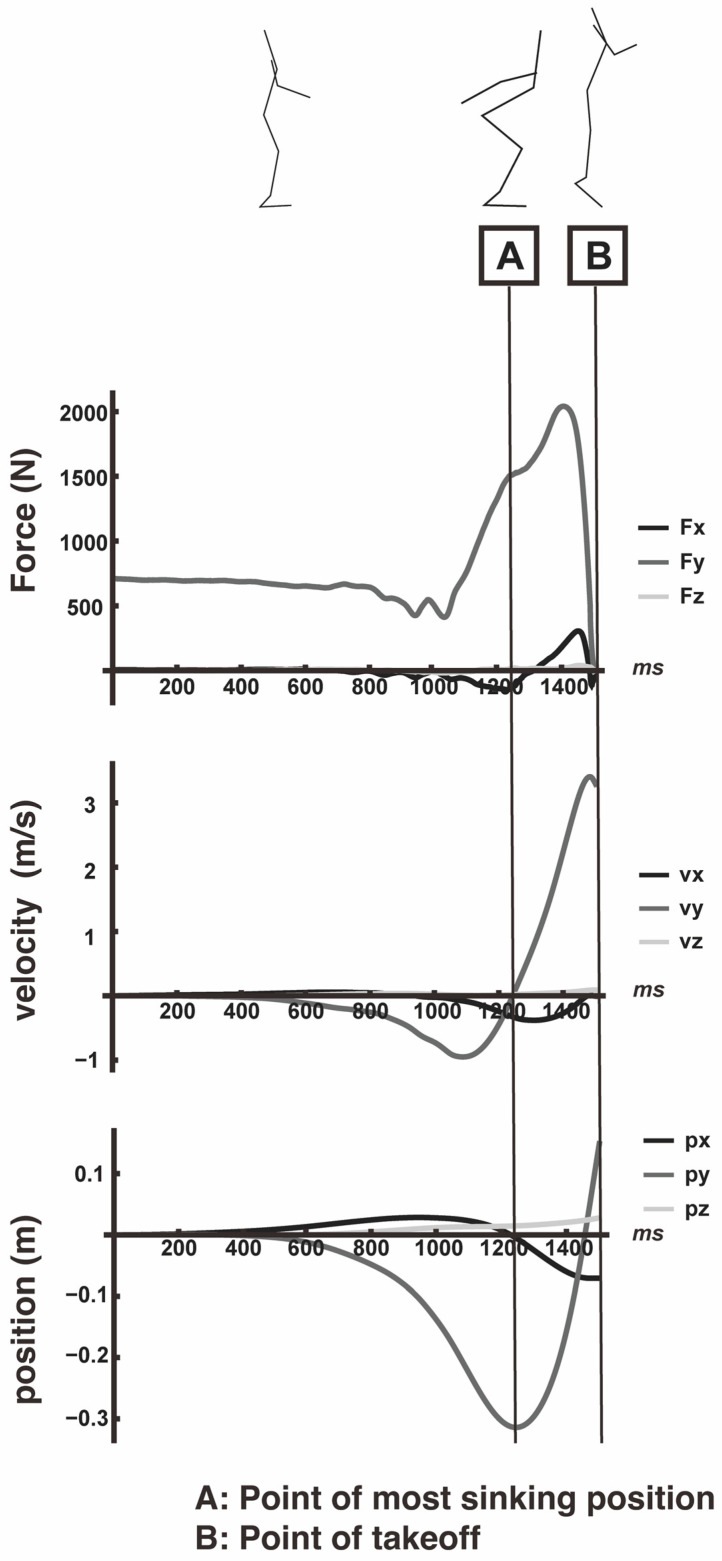
A typical example of analyzed data (above: each component of 3D force, middle: each component of 3D velocity, and below: each component of 3D position). A typical example of each variable used in the analysis. The most vertical sink point (A, t_0_) and takeoff point (B, t_1_) were calculated as the characteristic points common to all trials.

The trapezoidal rule was used for all of the numerical integrations. A typical example of the time-series data for the acceleration, velocity, and position vectors in each axis direction is shown in Figure 2.

#### 2.5.2. Position of the Center of Gravity in the Air, at the Highest Point of Arrival, and at Landing

The takeoff and landing time points coincided with the falling and rising points of the force data (e.g., Figure 1B: t_1_ and t_2_). These values are the coordinates of the xz plane of the cop in each trial but were converted to coordinates by subtracting the initial value (p_0_) from the following equations. As gravity is the only force acting on the center of gravity during the dwell time, the center of gravity during the dwell time is in constant acceleration movement in the y-axis direction and in constant velocity movement in the x- and z-axis directions (wave line in Figure 1A).

(5) $$p_{t1}=\left({cop}_{x\left(t1\right)},\;{cop}_{z\left(t1\right)}\right)-\left({cop}_{x\left(0\right)},\;{cop}_{z\left(0\right)}\right).$$

(6) $$p_{t2}=\left({cop}_{x\left(t2\right)},\;{cop}_{z\left(t2\right)}\right)-\left({cop}_{x\left(0\right)},\;{cop}_{z\left(0\right)}\right).$$

#### 2.5.3. Jumping Height

The jumping height was calculated according to the following equation using the y-axis velocity at takeoff. This value corresponds to the maximum value in the y-axis direction in Equation (7).

(7) $$h=\frac{{v^{2}}_{y\left(t_{1}\right)}}{2g_{y}}.\;$$

#### 2.5.4. Quantification of Each Point by Information Entropy

Using the x-z plane coordinates of the position vector at takeoff and landing for each trial and the coordinates of the 3D velocity vector at takeoff, entropy was calculated as an index for evaluating the variability of the vertical jump. All variables included all of the coordinates for each condition, and entropy was calculated using methods described in previous studies [17,18]. Each coordinate at each time point was encoded into a square or cube of 0.1 m per side, and entropy was calculated by computing the probabilities [17,18]. The method for determining the bin width was set independently in this study to ensure the accuracy of its analysis for the center-of-gravity trajectory of whole-body movements, as in previous studies on hand movements [17,18], because there were no previous studies that dealt with whole-body movements such as those in this study. This entropy was calculated using $H_{1}(X)\equiv \underset{a\to 1}{\lim}H_{a}\left(X\right)=\sum P_{i}\log_{2}\left(1/P_{i}\right)$, where $P_{i}$ is the frequency distribution of data points in bin *i*. The limiting value of $H_{a}$ as α→1 is Shannon entropy ([4,25]). In other words, in this analysis, if all coordinates are in the same square or cube part (encoded with the same value), the value of the information entropy is zero [17,18].

#### 2.5.5. Scalar Quantity of 3D Velocity Vector and Its Angle of Deviation from the Vertical Direction

The amount of scalar ($\left\|v_{t}\right\|$, Equation (8)) was calculated from the time-series 3D velocity vector data from the most vertical sink point ($t_{0}$ in Figure 2) to the takeoff point ($t_{1}$ in Figure 2). To calculate the angle of deviation from the most vertical sink point ($t_{0}$) to the takeoff point ($t_{1})$, which is the inner product of the ***v*** vector and the ***k*** vector, Equation (9) can be used.

(8) $$\left\|v_{t}\right\|=\sqrt{{{v_{x}}^{2}}_{t}+{{v_{y}}^{2}}_{t}+{{v_{z}}^{2}}_{t}},\;\left(t=t_{0}\sim t_{1}\right).\;$$

(9) $$v_{{ang}_{\left(t\right)}}=\cos^{-1}\frac{v_{t}\cdot k}{\left\|v_{t}\right\|},\;k=\left(0,\;1,\;0\right),\;\left(t=t_{0}\sim t_{1}\right).\;$$

### 2.6. Statistical Analysis

The results for the quantities of jumping height and 3D velocity vector scalar and their angle of deviation from the vertical direction are shown as mean values and standard deviations for all participants. A *t*-test was used to compare the values between the conditions. Entropy, which indicates the variability at each time point, is shown for each condition as values at the point of takeoff and landing calculated from the two-dimensional (x-z plane) position vector and values calculated from the three-dimensional velocity vector. Mathematica (Version 12.3.1.0; Wolfram Research, IL, USA) was used for statistical analysis, with a statistical significance level of *p* < 0.05.

## 3. Results

### 3.1. Jump Height of Vertical Jump

The adjusted condition had a significantly lower jump height than the normal condition (Nc: 0.48 ± 0.08 m, Ac: 0.45 ± 0.08 m, *t* (198) = 2.92, and *p* < 0.01). All participants tended to that the highest jump height recorded in the adjusted condition did not exceed the lowest jump height recorded in the normal condition. As the jumping height is determined by the velocity in the y-axis direction at the time of takeoff (Equation (8)), it can be inferred that the velocity at takeoff is lower in the adjusted condition than in the normal condition. In fact, the y-axis velocity at ground release was significantly smaller in the adjusted condition than in the normal condition (Nc: 2.92 ± 0.24 m/s, Ac: 2.81 ± 0.23 m/s, *t* (198) = 3.14, and *p* < 0.01).

### 3.2. Accuracy of Vertical Jump Measured by Entropy at Each Time Point

**Figure 3:**
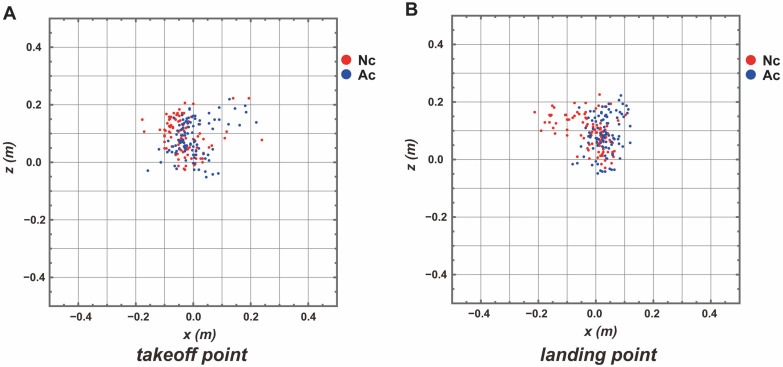
Xz coordinates of cop for takeoff and landing for each condition. (**A**) The xz coordinates of the center of gravity for each condition (red: normal condition; blue: adjusted condition) at the takeoff point. (**B**) The xz coordinates of the center of gravity for each condition (red: normal condition; blue: adjusted condition) at the landing point.

Figure 3 shows the two-dimensional coordinates (x-z plane) of the center of gravity at landing and takeoff. To quantify the variation in these coordinates, entropy was calculated using the method described in previous studies [17,18]. Consequently, the values at the time of landing were lower under the adjusted condition than under the normal condition (landing—Nc: 2.63; Ac: 2.49). In addition, when comparing the values at takeoff and landing, the values from takeoff to landing were smaller under the adjusted condition than under normal condition (takeoff—Nc: 2.69; Ac: 2.68). The entropy of the 3D velocity vector at the takeoff point was lower in the adjusted condition than in the normal condition (Nc: 5.94; Ac: 5.69).

### 3.3. Scalar Amount and Angle of Deviation from Vertical of 3D Velocity Vector

**Figure 4:**
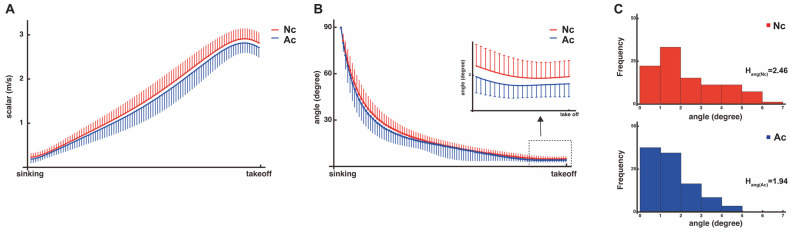
The performance variables for each condition. All of the performance variables for each condition contained data from 100 trials. (**A**) The mount of velocity scalar from the most vertical sink point to the takeoff point (red: normal condition, mean + SD; blue: adjusted condition, mean − SD). (**B**) The angle of deviation from the most vertical sink point to the takeoff point (red: normal condition, mean + SD; blue: adjusted condition, mean − SD). (**C**) A histogram of the angle at the takeoff point (red: normal condition; blue: adjusted condition). In the Ac, the values were clustered around 0° compared with the Nc. When entropy was calculated for a bin width of 1°, the value was smaller for Ac than for Nc.

The mean scalar of the 3D velocity vector for all participants from the most vertical sink point to the takeoff point is shown in Figure 4A. At the takeoff point, the values were significantly smaller for the adjusted condition than for the normal condition (Nc: 2.93 ± 0.24 m/s, Ac: 2.82 ± 0.23 m/s, *t* (198) = 3.17, and *p* < 0.01). The mean angle of deviation from the vertical direction of the 3D velocity vector for all participants from the most vertical sink point to the takeoff point is shown in Figure 4B. At the takeoff point, the values were significantly smaller for the adjusted condition than for the normal condition (Nc: 2.42 ± 1.58-degree, Ac: 1.58 ± 1.11-degree, *t* (198) = 4.37, and *p* < 0.01).

## 4. Discussion

### 4.1. Confirmation of the Speed and Accuracy Tradeoff in Whole-Body Movements with Aerial Phases

In this study, vertical jumps were performed under two conditions, normal and adjusted conditions, that required control of the landing position. The core results of this study are as follows: in both conditions, the jump was made with maximum effort, but the entropy of the 3D velocity vector at the point of takeoff was smaller in the adjusted condition than in the normal condition; the entropy of the 2D position vector was reduced (increased accuracy) from the point of takeoff to the point of landing, and the jump height was reduced. As the jumping height is determined by the velocity at takeoff in the vertical direction, it is understandable that an increase in accuracy and a decrease in velocity occurred under the adjusted condition. This supports the results of Yamada et al. [16] and suggests that even in full-body movements such as the vertical jump, the tradeoff between speed and accuracy observed in hand movement experiments [1,2,3] is valid within a certain period. Furthermore, the conventional speed and accuracy tradeoff is a relationship derived from the average resultant features of the movement, dealing with repetitive or discrete hand movements [1,2,3]. Considering this, the results of this study suggest that this relationship can occur even in single discrete whole-body movements that require approximately 1 s to complete, and confirmation of this phenomenon is crucial. This suggests that the demand for control of the landing position may have resulted not only in a feedforward, preplanned behavior acting on the movement, but also in some feedback behavior, resulting in position adjustment. Furthermore, in this study, simply verbally instructing participants to increase the accuracy of their landings and making them more aware of their accuracy than usual led to a decrease in leaping height and an increase in accuracy, an interesting result. It would be valuable to be able to derive changes in performance in this manner, rather than examining changes in performance with varied precision due to physical constraints (e.g., changes in target size) as typified in Fitts’s study [3].

### 4.2. Mechanism behind the Speed–Accuracy Tradeoff

Next, we focused on the characteristic that the landing position is determined when the center of gravity becomes a parabolic motion in the air of the vertical jump and leaves the ground [16] to investigate the mechanism by which this tradeoff phenomenon occurs. That is, the analysis was conducted with the idea that a movement adjustment between the start of the movement and takeoff from the ground caused a change in the accuracy of the landing. Consequently, both the mean scalar and mean angle of deviation from the vertical direction of the 3D velocity vector between the most vertical sink point and the takeoff point were significantly smaller in the adjusted condition than in the normal condition. Histograms of the angles at the takeoff point show that values were clustered around 0° in the adjusted condition compared with the normal condition (Figure 4C). When entropy was calculated using a bin width of 1°, the values were smaller under the adjusted condition (Ac: 1.94; Nc: 2.46) compared to under the normal condition (Nc). These results indicate that the speed and direction of the exerted velocity vector were adjusted during this time under the adjusted condition. Furthermore, the entropy of the 3D velocity vector at the takeoff point was lower in the adjusted condition than in the normal condition at the landing point, indicating that the variation in the velocity vector (magnitude and direction) was also adjusted. It was then shown that the value of entropy calculated from the 2D position vectors of takeoff and landing was more greatly reduced in the adjusted condition than in the normal condition (Nc—takeoff: 2.69, landing: 2.63; Ac—takeoff: 2.68, landing: 2.49).

Based on previous studies [17,18] that calculated the entropy of each time interval toward the target point using a tapping task similar to Fitts and that discussed information processing from the process of changing the value, the result that the value of the entropy of the adjusted condition decreased from the takeoff point to the landing point (the direction in which time progressed) is supported and more information processing (a decrease in the amount of information) is considered to have taken place. This entropy reduction in hand movements toward a target is thought to be coordinated by the movement associated with feedback control [26], in which the position–acceleration phase diagram becomes N-shaped, as reported in previous studies [17,18]. However, in vertical jumps such as those in the present study, since the movement involves an aerial phase after takeoff, the final landing position is determined by the coordination of the movement before takeoff, which can be interpreted as essentially different from the entropy reduction in hand movements that can be adjusted during the course of the trajectory.

**Figure 5:**
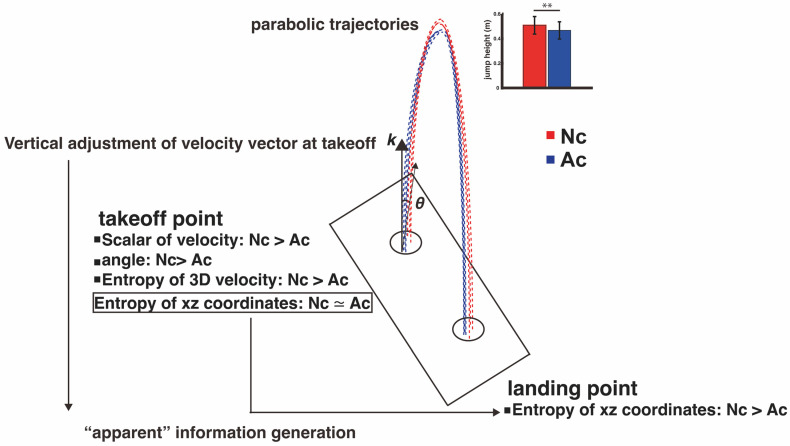
A conceptual diagram of each parameter related to position adjustment in a vertical jump. The wavy lines show a conceptual diagram of the trajectory of the position coordinates for each condition (red: normal condition [Nc]; blue: adjusted condition [Ac]). The takeoff point parameters associated with this trajectory differ between conditions. ** Significant at *p* < 0.01.

Figure 5 shows a conceptual diagram based on the parameters of the conditions calculated from the actual analysis, which may influence the adjustment of the landing point in a vertical jump. The diagram demonstrates that the trajectories, represented by wavy lines leading up to the landing point, are determined by the initial velocity vectors at takeoff. The modification of the landing trajectory is reflected in the difference between the entropy at takeoff and the entropy at landing, computed as “apparent” information generation. As mentioned previously, entropy reduction in hand movements can be achieved using feedback control from the latter half of the trajectory to capture the target accurately. However, the center of gravity becomes a parabolic trajectory after takeoff, and such feedback control is not available. Thus, the trajectory is predetermined in a feedforward manner by setting the initial velocity direction at takeoff, enabling adjustment of the landing point. Thus, the application of entropy analysis to movement analysis is very effective for quantifying information processing during movement. Notably, this control process is evaluated as “apparent” information generation during the aerial phase.

### 4.3. An Enhanced Movement Model for Vertical Jumps

**Figure 6:**
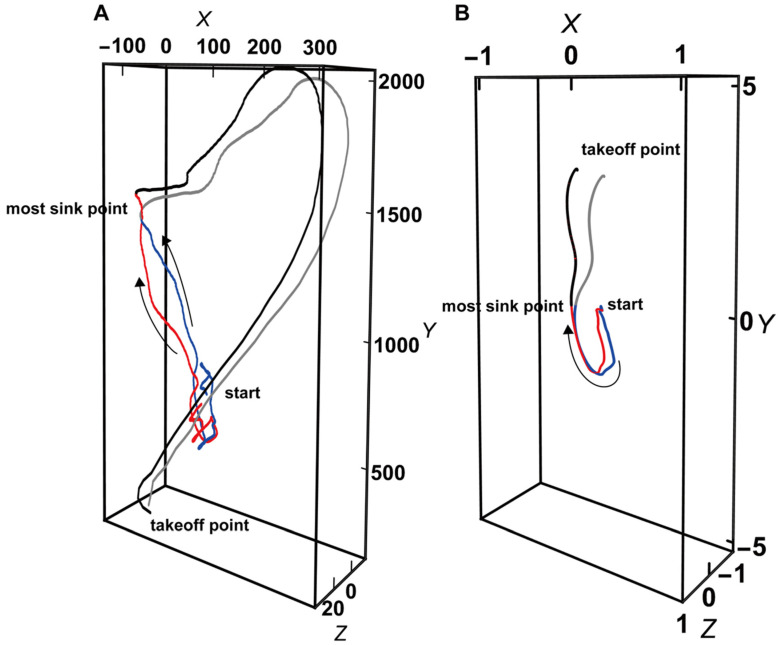
A typical example of a 3D force vector waveform (**A**) and a 3D velocity vector waveform (**B**). Classified from the start of the movement to the most vertical sink point (red line: normal condition; blue line: adjusted condition) and from the most vertical sink point and to the takeoff point (black line: normal condition; gray line: adjusted condition).

This study reveals the characteristic results of 3D velocity vectors from the most vertical sink point to the takeoff point as the mechanism by which the decrease in jump height and increase in landing position accuracy in the adjusted condition occurred. This section concludes by discussing the control of the movement phase up to the most vertical sink point and derives points that should be clarified in the future. Yamada et al.’s study [16], on which this study is based, made the following important points: Comparing the adjusted and normal conditions, it is necessary to examine the final value of the integral from the start of movement in the y-axis direction to the takeoff with respect to the decrease in jumping height, which is reduced from the normal condition, and the process leading to that value. For this purpose, instead of qualitatively comparing the magnitude of the waveforms of both conditions, we compared them from three perspectives of direction and duration of action. As mentioned at the beginning of this study, the following characteristic results were obtained regarding the direction and 3D velocity scalar from the most vertical sink point to the takeoff point: In the adjusted condition, the landing position was accurately controlled by the takeoff more in the vertical direction and with a more suppressed scalar amount. However, the time from the most vertical sink point to the takeoff point was examined, and there was no significant difference between conditions (Nc: 281 ± 46 ms, Ac: 289 ± 62 ms, *t* (198) = 0.97, and *p* = 0.34). That is, there was no significant difference in movement time between the conditions during this phase. This result suggests that there was no detailed motion coordination during this movement phase and that some control may have taken place prior to this phase. Therefore, the time to the most vertical sink point was calculated by setting the point of the starting movement from rest at ±10% of each participant’s mass, and that time was significantly longer in the adjusted condition than in the normal condition (Nc: 815 ± 188 ms, Ac: 879 ± 222 ms, *t* (198) = 2.21, and *p* < 0.05). Qualitative observation of this movement phase to the most vertical sink point shows that the magnitude of the y-axis force exerted gradually increases, and the velocity vector switches direction and accelerates upward in the first half of the phase (Figure 6). That is, in the adjusted condition, the final position adjustment may have occurred by spending more time in this phase compared to in the normal condition. Therefore, it could be explained that during this phase, the participant was moving as follows: perceiving the current direction of movement while exerting force and vertically adjusting their speed in the first half of the vertical acceleration. Yamada et al.’s [16] qualitative analysis using typical examples of each condition suggested the possibility that V-F conversion (functionalization of velocity and force) was performed during the sink movement phase. This study quantified and demonstrated this possibility based on the relationship between action times. In future research, it will be necessary to further investigate the essence of sports movements by comparing and modeling the movement phase in which this V-F conversion occurs.

## 5. Conclusions

In the dynamic task of vertical jumping, typically characterized by maximal exertion, our introduction of entropy calculations has yielded a profound insight into the mechanisms of precision adjustment. The target of controlling the landing position resulted in a marked improvement in accuracy, as evidenced by the adoption of more vertical takeoff angles, a reduced magnitude of velocity, and a reduced velocity. More crucially, the application of entropy calculations to this motor task has unveiled a nuanced facet of performance enhancement: a decrease in entropy at the point of takeoff signals a heightened level of control and predictability in the execution of the jump. This entropy reduction, reflecting a concentrated effort to minimize variability, substantiates the complexity of the speed–accuracy tradeoff in a rapid, whole-body movement. It demonstrates that entropy is not merely a theoretical construct but a practical tool that quantitatively dissects the information processing involved in fine-tuning movements for increased accuracy. Our findings advocate the integration of entropy calculations into motor control research, proposing that this methodological advancement can significantly refine our understanding of precision control in physical movements.

## Data Availability

The datasets and programming codes generated or analyzed in the current study are available from the corresponding author upon reasonable request.
